# ASAS-NANP symposium: mathematical modeling in animal nutrition: training the future generation in data and predictive analytics for sustainable development. A summary of the 2024 symposium

**DOI:** 10.1093/jas/skaf452

**Published:** 2025-12-27

**Authors:** Luis O Tedeschi, Hector M Menendez III

**Affiliations:** Department of Animal Science, Texas A&M University, College Station, TX 77843-2471; South Dakota State University West River Research and Extension Center, Rapid City, SD 57701

**Keywords:** Modeling, Simulation, Conference, Learning, Education, Nutrition

## Introduction

The livestock sector stands at a computational crossroads. While traditional mechanistic models have served animal nutrition for over a century (France and Kebreab, 2008; Tedeschi and Fox, 2020), the convergence of unprecedented data availability, artificial intelligence (AI) capabilities, and emerging quantum computing (QC) architectures is fundamentally reshaping what is computationally feasible in understanding complex biological systems. This transformation extends beyond incremental improvements in prediction accuracy, representing a paradigm shift in how we conceptualize, model, and optimize livestock production systems for sustainability and efficiency. The challenge facing animal scientists is not merely adopting new computational tools, but developing the theoretical frameworks and practical skills needed to integrate these technologies meaningfully into biological research and application.

Recognizing this inflection point, the Nutrition Modeling Committee of the National Animal Nutrition Program (NANP; https://animalnutrition.org) has strategically evolved its training initiatives to prepare future generations for this computational revolution, from basic modeling techniques to advanced methods, and more recently, has begun introducing quantum aspects into mathematical modeling. Over seven years (2018 to 2024), these symposia have progressed from foundational modeling principles to cutting-edge applications, consistently drawing 60–100 participants annually and generating peer-reviewed publications that have been cited more than 319 times (Tedeschi et al., 2021; Tedeschi et al., 2023; Tedeschi and Menendez III, 2025). The sustained engagement reflects both the quality of training and the urgency with which the animal science community seeks to master advanced analytical methods.

Feedback from participants in the 2024 symposium further underscores the relevance and effectiveness of this training effort. Survey responses indicated strong acceptance of the symposium content, with 95% of respondents rating the material as at least moderately useful and 50% rating it as very or extremely useful to their research or professional activities. Participants also reported measurable gains in self-assessed understanding and use of predictive technologies, modeling software, and data management strategies following the symposium, alongside high overall satisfaction with content quality (mean = 3.88/5), subject matter coverage (mean = 4.08/5), and overall experience (mean = 3.83/5), reinforcing the symposium’s role as a meaningful capacity-building initiative for a diverse audience spanning students, early-career scientists, and established professionals.

## Bridging Classical Modeling with Frontier Computing: The 2024 Symposium

The seventh symposium, held in Calgary, Canada, on July 21, 2024, addressed the most pressing computational frontiers in animal science by integrating system dynamics (SD), advanced machine learning (ML) architectures, and quantum computing paradigms. This thematic focus reflects broader scientific trends: QC has emerged as a transformative technology for solving complex optimization problems that were previously intractable on classical computers (Preskill, 2018), with early agricultural applications demonstrating potential for supply chain optimization and resource allocation (Tedeschi, 2026). Simultaneously, AI continues revolutionizing agricultural management through computer vision, predictive analytics, autonomous decision-making systems, and supply chain management (Liakos et al., 2018; Sharma et al., 2020). The integration of life cycle assessment (LCA) with biological modeling addresses the critical need for comprehensive environmental sustainability metrics in livestock production (Baldini et al., 2017).

The 2024 symposium featured two 40-min presentations and three 90- to 110-min hands-on training workshops addressing, in a progressive sequence, SD modeling as operational “flight simulators” for livestock production systems, construction of robust supervised ML regression pipelines, time series techniques for predictive livestock management, comprehensive AI frameworks for farm management transformation, mathematical modeling contributions to environmental LCA, QC applications in animal nutrition, and agent-based modeling (ABM) for swine nutrition systems. This program represents the culmination of progressive development across previous symposia. Early events (2018–2019) established foundational modeling concepts and systems thinking frameworks (Nicholson et al., 2019; Tedeschi, 2019). Intermediate symposia (2020–2021) introduced hybrid intelligent mechanistic models (HIMM), precision livestock farming (PLF), and graphical data analytics methodologies (Gerrits et al., 2021; Morota et al., 2021; Stephens, 2021; Wang et al., 2021; Jacobs et al., 2022; Menendez et al., 2022; Tedeschi, 2022). The 2022 and 2023 symposia advanced practical applications through big data, ABM, digital twins, synthetic database generation, and satellite-based decision support (Brennan et al., 2023; Kaniyamattam and Tedeschi, 2023; Muñoz-Tamayo and Tedeschi, 2023; Brown-Brandl and Tao, 2025; Fernandes and Tedeschi, 2025; Oviedo-Rondón, 2025; Tedeschi, 2025).

The 2024 symposium extends this trajectory by addressing implementation frameworks that illustrate how modern animal nutrition modeling is evolving from isolated analytical tools into an integrated computational ecosystem that spans biological realism, data-driven inference, decision support, and frontier computing. The papers were organized to reflect a deliberate progression, from empirical and enabling methods to increasingly mechanistic, hybrid, and individual-based modeling approaches, culminating in frontier computational paradigms that truly advance the industry and producer application of animal nutrition models. Together, these contributions demonstrate complementary pathways for addressing complexity, uncertainty, and scalability in livestock systems that has resulted from the seemingly non-stop transformation over the past decade.

Tulpan et al. (2026) operationalize supervised learning by presenting a transparent, open-source regression pipeline for modeling livestock data. Their case-study-driven approach demystifies ML workflows, emphasizing preprocessing, diagnostics, interpretability, and reproducibility. By prioritizing explainability and open access, this paper addresses a critical adoption barrier identified across multiple symposium contributions: the need for ML tools that are not only accurate but scientifically defensible and accessible to researchers, students, and practitioners alike. Building on this foundation, Peter et al. (2026) focus on the temporal dimension of livestock systems by reviewing time-series and ML approaches for dynamic prediction. Their paper clarifies when classical statistical methods (e.g., Autoregressive Integrated Moving Average with eXogenous variables—ARIMAX, Kalman filtering) remain appropriate and when deep learning architectures (e.g., Recurrent Neural Networks—RNN, transformers) offer advantages for handling nonlinear, autocorrelated production data. This contribution reinforces the idea that model choice must be driven by data structure, biological context, and forecasting objectives, rather than methodological novelty. As livestock production systems seek not only to optimize livestock nutritional management but to forecast and alter individual energy and protein demands, time-aware (i.e., real-time) modeling has never been a more critical requirement for predictive nutrition, health monitoring, and adaptive management.

Condotta and Tedeschi (2026) extend this systems-level perspective by reviewing how AI is reshaping livestock management across health, reproduction, behavior, and nutrition. Their contribution situates AI not as a replacement for biological understanding, but as an enabling layer that extracts signal from increasingly dense and heterogeneous data streams generated by precision livestock technologies. Notably, the paper highlights persistent barriers—data quality, interpretability, infrastructure, and ethical considerations—that reinforce the symposium’s recurring theme: predictive power alone is insufficient without trust, transparency, and human-centered design. This perspective aligns with the broader evolution of PLF, in which sensor technologies, computer vision, and AI have progressed from passive monitoring tools to integrated decision-support systems that increasingly interface with mechanistic and hybrid nutrition models (Tedeschi et al., 2026). Menendez III et al. (2026) bridge theory and practice by introducing SD–based “flight simulators” as operational interfaces for complex nutrition and management models. These simulators translate abstract equations into experiential learning environments, enabling stakeholders to test scenarios, gather feedback, and anticipate unintended consequences. Menendez III et al. (2026) highlight that these “microworlds” allow users to make mistakes regarding the rapidly evolving requirements of precision livestock technology, nutrition models, and cyberinfrastructure, without having to bear the consequences of failure. Thus, in a non-stop period of transformation, this work directly addresses a longstanding limitation in animal nutrition modeling: the difficulty of translating sophisticated models into actionable insights to avoid unintended consequences that erode the livestock industry’s sustainability and efficiency while increasing costs. By embedding Systems Thinking into user-facing tools, flight simulators serve as the connective tissue between mechanistic models, AI-driven analytics, and real-world decision-making.

Cadéro et al. (2026) provide a foundational anchor by demonstrating how mathematical modeling strengthens LCA by mechanistically representing animal performance, nutrient excretion, and emission processes. Their review shows that traditional survey-based or emission-factor LCA approaches lack the resolution needed to capture interactions among nutrition, genetics, management, and environment. By embedding nutritional and process-based models within the LCA framework, the authors elevate LCA from a descriptive accounting exercise to a predictive, scenario-driven decision support tool. This paper establishes environmental sustainability as a modeling outcome that must be explicitly coupled to biological processes rather than appended post hoc. Rahimifar et al. (2026) advance individual-level representation by developing an ABM for swine nutrition and illustrate how such approaches can support systems designed for sustainable livestock production. By explicitly simulating individual pigs rather than an “average animal,” the model captures biological variability in growth, nutrient requirements, and environmental impact. This work exemplifies a broader shift emphasized throughout the symposium: moving from population-level approximations toward individualized, stochastic representations that better align with precision feeding and sustainability goals. The ABM framework also naturally complements both LCA and AI approaches by generating rich, bottom-up data streams.

Finally, Tedeschi (2026) explores the potential role of QC in agricultural and animal sciences, framing QC as a long-term but potentially transformative extension of existing computational paradigms. Rather than promising near-term solutions, the paper situates QC realistically within developmental timelines and emphasizes hybrid quantum–classical approaches. Its inclusion in the symposium underscores a forward-looking message: as biological models grow in dimensionality and optimization complexity, classical computing—even when augmented by AI—may encounter fundamental limits that warrant exploration of alternative architectures.

Taken together, the 2024 symposium papers reveal a coherent progression: mechanistic biological models ground predictions; AI and machine learning extract patterns and enable scalability; SD and flight simulators translate complexity into insight for industry adopters; agent-based and time-series methods capture variability and dynamics; LCA frameworks connect production to environmental outcomes; and quantum computing represents a speculative but strategic horizon. Rather than competing approaches, these methodologies form a layered modeling ecosystem in which each component addresses specific limitations of the others. The unifying message of the 2024 symposium is that advancing animal nutrition modeling is no longer about choosing between mechanistic or data-driven approaches, but about orchestrating multiple computational paradigms within biologically informed, decision-oriented frameworks. This synthesis marks a maturation point for the NANP Modeling Committee’s long-term vision and sets the stage for future efforts focused on integration, interoperability, and real-world implementation to enhance modeling for the digital age.

## Abbreviations:

ABM: agent-based modeling
AI: artificial intelligence
ASAS: American Society of Animal Science
HIMM: hybrid intelligent mechanistic model
LCA: life cycle assessment
ML: machine learning
NANP: National Animal Nutrition Program
NIFA: National Institute of Food and Agriculture
PLF: precision livestock farming
QC: quantum computing
SD: system dynamics
USDA: United States Department of Agriculture

## References

[skaf452-B1] Baldini C. , Gardoni D., Guarino M. 2017. A critical review of the recent evolution of Life Cycle Assessment applied to milk production. J. Cleaner Prod. 140:421–435. 10.1016/j.jclepro.2016.06.078

[skaf452-B2] Brennan J. , Menendez, III H. M., Ehlert K., Tedeschi L. O. 2023. ASAS-NANP symposium: mathematical modeling in animal nutrition—making sense of big data and machine learning: how open-source code can advance training of animal scientists. J. Ani. Sci. 101:skad317. 10.1093/jas/skad317 PMC10664406 37997926

[skaf452-B3] Brown-Brandl T. , Tao J. 2025. ASAS-NANP symposium: mathematical modeling in animal nutrition: harnessing real-time data and digital twins for precision livestock farming. J. Anim. Sci. 103:skaf138. 10.1093/jas/skaf138 PMC12351255 40319358

[skaf452-B4] Cadéro A. , Tedeschi L. O., Garcia-Launay F. 2026. ASAS-NANP symposium: mathematical modeling in animal nutrition: contributions of mathematical modeling to life cycle assessment to support environmental sustainability of animal production. J. Anim. Sci. 104:skaf442. https://doi.org/10.1093/jas/skaf442 PMC12863956 41410510

[skaf452-B5] Condotta I. C. F. S. , Tedeschi L. O. 2026. ASAS-NANP symposium: mathematical modeling in animal nutrition: revolutionizing animal farming with artificial intelligence: Trends, challenges, and opportunities. J. Anim. Sci. 104:skaf441. https://doi.org/10.1093/jas/skaf441 PMC12875599 41410516

[skaf452-B6] Fernandes M. H. M. R. , Tedeschi L. O. 2025. ASAS-NANP symposium: mathematical modeling in animal nutrition: application of modeling innovations to support satellite remote sensing for sustainable grazing cattle management. J. Anim. Sci. 103:skaf137. 10.1093/jas/skaf137 PMC12351259 40319360

[skaf452-B7] France J. , Kebreab E. 2008. Mathematical modelling in animal nutrition. Wallingford, UK: CABI Publishing.

[skaf452-B8] Gerrits W. , Schop M., de Vries S., Dijkstra J. 2021. ASAS-NANP symposium: digestion kinetics in pigs: the next step in feed evaluation and a ready-to-use modeling exercise. J. Anim. Sci. 99(2):1–8. 10.1093/jas/skab020 PMC7904037 33626147

[skaf452-B9] Jacobs M. , Remus A., Gaillard C., Menendez, III H. M., Tedeschi L. O., Neethirajan S., Ellis J. L. 2022. ASAS-NANP symposium: mathematical modeling in animal nutrition: limitations and potential next steps for modeling and modelers in the animal sciences. J. Anim. Sci. 100(6):1–15. 10.1093/jas/skac132 PMC9171330 35419602

[skaf452-B10] Kaniyamattam K. , Tedeschi L. O. 2023. ASAS-NANP symposium: mathematical modeling in animal nutrition: agent-based modeling for livestock systems: the mechanics of development and application. J. Anim. Sci. 101:skad321. 10.1093/jas/skad321 PMC10664392 37997925

[skaf452-B11] Liakos G. K. , Busato P., Moshou D., Pearson S., Bochtis D. 2018. Machine learning in agriculture: a review. Sensors (Basel). 18(8):2674. 10.3390/s18082674 PMC6111295 30110960

[skaf452-B12] Menendez H. M. III , Brennan J. R., Gaillard C., Ehlert K., Quintana J., Neethirajan S., Remus A., Jacobs M., Teixeira I. A. M. A., Turner B. L. et al. 2022. ASAS-NANP symposium: mathematical modeling in animal nutrition: opportunities and challenges of confined and extensive precision livestock production. J. Anim. Sci. 100(6):1–19. 10.1093/jas/skac160 PMC9171331 35511692

[skaf452-B13] Menendez H. M. III , Chang Y., Palmer E., Manna F. L., Moreno E. R. V., Parsons I., Turner B. L., Rekabdarkolaee H. M., Husmann A. L., Tedeschi L. O. 2026. ASAS-NANP symposium: mathematical modeling in animal nutrition: the importance of animal science flight simulators to enhance the competitiveness and sustainability of livestock production. J. Anim. Sci. 104:skaf440. doi: 10.1093/jas/skaf440 PMC12893780 41410526

[skaf452-B14] Morota G. , Cheng H., Cook D., Tanaka E. 2021. ASAS-NANP symposium: prospects for interactive and dynamic graphics in the era of data-rich animal science. J. Anim. Sci. 99(2):1–17. 10.1093/jas/skaa402 PMC7904041 33626150

[skaf452-B15] Muñoz-Tamayo R. , Tedeschi L. O. 2023. ASAS-NANP symposium: mathematical modeling in animal nutrition: the power of identifiability analysis for dynamic modeling in animal science—a practitioner approach. J. Anim. Sci. 101:skad320. 10.1093/jas/skad320 PMC10664400 37997927

[skaf452-B16] Nicholson C. F. , Peixoto Simões A. R., Van Amburgh M. E., LaPierre P. A. 2019. ASN-ASAS symposium: future of data analytics in nutrition: modeling complex problems with system dynamics: applications in animal agriculture. J. Anim. Sci. 97(5):1903–1920. 10.1093/jas/skz105 PMC6488312 30923803

[skaf452-B17] Oviedo-Rondón E. 2025. ASAS-NANP symposium: mathematical modeling in animal nutrition: overview of poultry nutrition modeling. J. Anim. Sci. 103:skaf140. 10.1093/jas/skaf140 PMC12351263 40319359

[skaf452-B18] Peter J. , Li M., Brennan J., Menendez H., Rekabdarkolaee H. M. 2026. ASAS-NANP symposium: mathematical modeling in animal nutrition: review of time series techniques and machine learning models for advancing livestock production. J. Anim. Sci. 104:skaf439. doi: 10.1093/jas/skaf439 PMC12863955 41410519

[skaf452-B19] Preskill J. 2018. Quantum computing in the NISQ era and beyond. Quantum. 2(1801.00862v3):79–20. 10.22331/q-2018-08-06-79

[skaf452-B20] Rahimifar A. , Kaniyamattam K., Wiegert J. G., Tedeschi L. O. 2026. ASAS-NANP symposium: mathematical modeling in animal nutrition: developing an agent-based model to calculate growth performance and predict nutrient requirements of growing-finishing pigs toward sustainability. J. Anim. Sci. 104:skaf443. 10.1093/jas/skaf443 PMC12924631 41410523

[skaf452-B21] Sharma R. , Kamble S. S., Gunasekaran A., Kumar V., Kumar A. 2020. A systematic literature review on machine learning applications for sustainable agriculture supply chain performance. Comput. Oper. Res. 119:104926. 10.1016/j.cor.2020.104926

[skaf452-B22] Stephens E. C. 2021. ASAS-NANP symposium: review of systems thinking concepts and their potential value in animal science research. J. Anim. Sci. 99(2):1–7. 10.1093/jas/skab021 PMC8631063 33626146

[skaf452-B23] Tedeschi L. O. 2019. ASN-ASAS symposium: future of data analytics in nutrition: mathematical modeling in ruminant nutrition: approaches and paradigms, extant models, and thoughts for upcoming predictive analytics. J. Anim. Sci. 97(5):1921–1944. 10.1093/jas/skz092 PMC6488328 30882142

[skaf452-B24] Tedeschi L. O. 2022. ASAS-NANP symposium: mathematical modeling in animal nutrition: the progression of data analytics and artificial intelligence in support of sustainable development in animal science. J. Anim. Sci. 100(6):1–11. 10.1093/jas/skac111 PMC9171329 35412610

[skaf452-B25] Tedeschi L. O. 2025. ASAS-NANP symposium: mathematical modeling in animal nutrition: synthetic database generation for non-normal multivariate distributions: a rank-based method with application to ruminant methane emissions. J. Anim. Sci. 103:skaf136. 10.1093/jas/skaf136 PMC12351256 40319357

[skaf452-B26] Tedeschi L. O. 2026. ASAS-NANP symposium: mathematical modeling in animal nutrition: quantum computing in agricultural sciences: from theory to reality. J. Anim. Sci. 104:skaf445. 10.1093/jas/skaf445 PMC12930474 41410533

[skaf452-B27] Tedeschi L. O. , Bureau D. P., Ferket P. R., Trottier N. L. 2021. ASAS-NANP symposium: mathematical modeling in animal nutrition: training the future generation in data and predictive analytics for sustainable development. A summary. J. Anim. Sci. 99(2):1–3. 10.1093/jas/skab023 PMC7904039 33626148

[skaf452-B28] Tedeschi L. O. , Fox D. G. 2020. The ruminant nutrition system: an applied model for predicting nutrient requirements and feed utilization in ruminants. Vol. 1. (3rd ed.) (3rd repr.). Dubuque, IA, USA: Kendall Hunt. First published in 2020 by XanEdu, Ann Arbor, MI. Available at https://he.kendallhunt.com/product/ruminant-nutrition-system-vol-1. Accessed on: February 7, 2025.

[skaf452-B29] Tedeschi L. O. , Guarnido-Lopez P., Menendez, III H. M., Seo S. 2026. Advancing precision livestock farming: integrating artificial intelligence and emerging technologies for sustainable livestock management. Anim. Biosci. 10.5713/ab.25.0289 PMC13057718 40808567

[skaf452-B30] Tedeschi L. O. , Menendez III H. M. 2025. ASAS-NANP symposium: mathematical modeling in animal nutrition: Training the future generation in data and predictive analytics for sustainable development. A summary of the 2023 symposium. J. Anim. Sci. 103: skaf141. 10.1093/jas/skaf141 PMC12317274 40319365

[skaf452-B31] Tedeschi L. O. , Menendez, III H. M., Remus A. 2023. ASAS-NANP symposium: mathematical modeling in animal nutrition: training the future generation in data and predictive analytics for sustainable development. A summary of the 2021 and 2022 symposia. J. Anim. Sci. 101:skad318. 10.1093/jas/skad318 PMC10664387 37997923

[skaf452-B32] Tulpan D. , Tedeschi L. O., Menendez, III H. M., Vieira R. A. M. 2026. ASAS-NANP symposium: mathematical modeling in animal nutrition: construction of supervised machine learning regression pipelines for livestock data modeling: a case study. J. Anim. Sci. 104.skaf444. https://doi.org/10.1093/jas/skaf444 PMC12863957 41437623

[skaf452-B33] Wang Z. , Shadpour S., Chan E., Rotondo V., Wood K., Tulpan D. 2021. ASAS-NANP symposium: applications of machine learning for livestock body weight prediction from digital images. J. Anim. Sci. 99(2):1–15. 10.1093/jas/skab022 PMC7904040 33626149

